# Zinc finger gene 217 (ZNF217) Promoted Ovarian Hyperstimulation Syndrome (OHSS) through Regulating E_2_ Synthesis and Inhibiting Thrombospondin-1 (TSP-1)

**DOI:** 10.1038/s41598-017-03555-6

**Published:** 2017-06-12

**Authors:** Junyu Zhai, Jiansheng Liu, Xiaoyue Cheng, Shang Li, Yan Hong, Kang Sun, Zi-Jiang Chen, Yanzhi Du, Weiping Li

**Affiliations:** 1 Center for Reproductive Medicine, Ren Ji Hospital, School of Medicine, Shanghai Jiao Tong University, Shanghai, 200135 China; 2 Shanghai Key Laboratory for Assisted Reproduction and Reproductive Genetics, Shanghai, 200135 China; 3 National Research Center for Assisted Reproductive Technology and Reproductive Genetics, The Key Laboratory for Reproductive Endocrinology of Ministry of Education, Shandong Provincial Key Laboratory of Reproductive Medicine, Center for Reproductive Medicine, Shandong Provincial Hospital, Shandong University, Jinan, 250021 China

## Abstract

Zinc finger gene 217 (ZNF217) is a candidate gene of polycystic ovary syndrome (PCOS) which is vulnerable to ovarian hyperstimulation syndrome (OHSS). However, the relationship between ZNF217 and OHSS is largely unknown. Our study demonstrated that ZNF217 was mainly distributed in the granulosa cells of rat ovary. Significantly higher expression of ovarian *ZNF217* was detected in OHSS rats, being consistent with serum 17β-estradiol concentration and ovarian aromatase. Moreover, OHSS rats also showed decreased ovarian *TSP-1* mRNA, an acknowledged VEGF signaling suppressor. The same changes were detected in human granulosa cells and follicular fluid. Thus, the increased ZNF217 and decreased TSP-1 may participate in OHSS onset. *In vitro* experiment revealed that ZNF217 positively regulated E_2_ synthesis through promoting *cAMP response element binding protein (CREB)* and thereby *CYP19A1* in KGN cells. Furthermore, ZNF217 negatively regulated TSP-1 in KGN cells while TSP-1 promoted claudin1 and inhibited nitric oxide (NO) in HUVECs and HAECs. Both of claudin1 and NO are responsible for the regulation of vascular permeability (VP). Therefore, we demonstrated that ZNF217 contributed to OHSS onset through promoting E_2_ synthesis and the increase of VP. Moreover, the increased ZNF217 and decreased TSP-1 provided new targets for the prevention or treatment of OHSS in the future.

## Introduction

Ovarian hyperstimulation syndrome (OHSS) is an iatrogenic and serious complication after ovarian stimulation. The ovarian enlargement, ascites, high level of 17 β-estradiol (E_2_) and high vascular permeability (VP) are the features of OHSS^1^. High serum E_2_ level on the day of human chorionic gonadotropin (hCG) administration is considered as a risk factor for the incidence of OHSS^2^. Thus, serum E_2_ plays an indispensable role in OHSS onset and some E_2_ inhibition drugs have been used in clinic to induce ovulation as well as preventing OHSS^3^.

Vascular endothelium growth factor (VEGF) promoted VP, another characteristic of OHSS, by binding to and phosphorylating VEGF receptor 2 (VEGFR2) in endothelial cells^4^. Estrogen receptor α (ERα) is a positive regulator of VEGF in MCF-7 cells^5^.

Zinc finger gene 217 (*ZNF217*) is a potential oncogene which located on chromosome 20q13.2^6^. The 8 zinc fingers inside it and the complex of ZNF217 and transcriptional co-repressor CoREST provide evidences for the role of ZNF217 as a transcription factor^7^. ZNF217 provides a selective advantage to cancer cells by inducing resistance to chemotherapy, in particular through interfering with survival pathways or deregulating apoptotic signals^8^. Previous study showed that ZNF217 and ERα proteins bound to each other in breast cancer cells while ERα positively regulated the expression of *VEGF* ^5^. Besides, ZNF217 promotes the ERα-dependent transcription of the downstream genes by enhancing the recruitment of ERα to its estrogen response elements (ERE)^9^. Thus, ZNF217 may also participate in the regulation of VEGF and OHSS. Moreover, *ZNF217* is a candidate gene of PCOS, regarded as a high risk factor of OHSS onset^10^. Thus, we suppose that ZNF217 is involved in the pathogenesis of OHSS^11, 12^. However, most previous researches about ovarian ZNF217 are related to poor patient survival in ovarian cancer^13^ or growth and survival of ovarian clear cell carcinoma (OCCCs)^14^. Therefore, the location of ZNF217 in normal ovaries as well as the relationship between ZNF217 and OHSS will still need to be studied.

Thrombospondin-1 (TSP-1) is a large matricellular glycoprotein which is distributed in different types of cells. It was first discovered in 1971 from α granules of platelet and took part in the promotion of platelet aggregation and related hemostatic functions^15^. Besides of thrombosis regulation, TSP-1 also participates in angiogenesis suppression by inhibiting the expression and downstream factors of VEGF which is an important inducer of high VP^16^. Furthermore, nitric oxide (NO) is of vital importance in VP regulation and TSP-1 has been found to function as an inhibitor of NO synthesis and signaling pathway after binding with one of its receptors CD47^17^. In a word, TSP-1 may also be involved in OHSS development through VP regulation. TSP-1 is regulated by ER^18^ while ZNF217 binds to ERα and strengthens its function. Moreover, the result of CHIP-seq in MCF-7 cell line indicated that ZNF217 bond to the promoter of *TSP-1* directly^19^. Therefore, TSP-1 may be regulated by ZNF217 in the ovary and is involved in OHSS onset.

Previous evidences have indicated the possible role of ZNF217 in the pathogenesis of OHSS. Therefore, our study investigated the distribution as well as the functions of ovarian ZNF217 in the development of OHSS.

## Results

### Localization of ZNF217 in rat ovary

**Figure 1:**
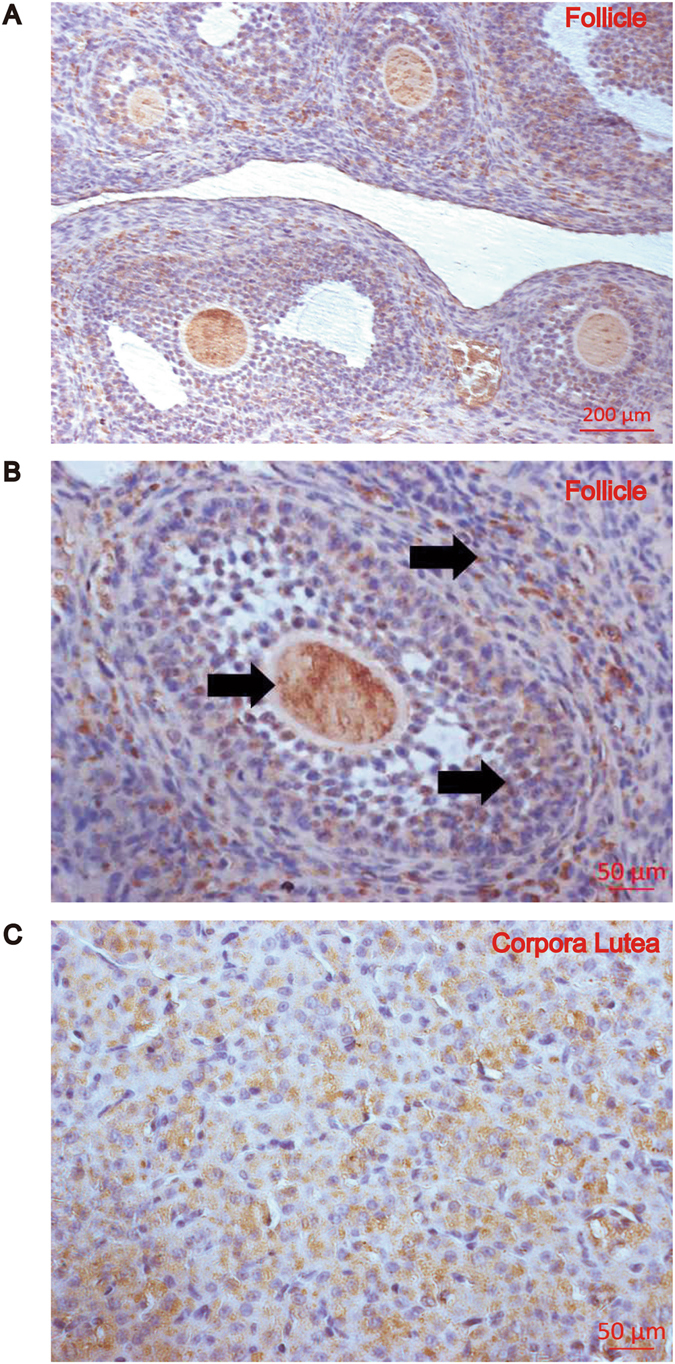
The distribution of ZNF217 in the ovaries of rats. (**A**,**B**) The location of ZNF217 in the follicles of control rats. Nuclei were counterstained with hematoxylin. (**C**) The expression of ZNF217 in the mature corpora lutea of control rats. Arrows showed the immunostaining of ZNF217.

ZNF217 appeared in the oocytes, granulosa cells and theca cells in the growing follicles of control rats (Fig. 1A,B) while immunostaining was mainly present in the luteinized granulosa cells of mature corpora lutea after ovulation (Fig. 1C). Highest expression level of ZNF217 was observed in the granulosa cells and luteal cells with immunostaining concentrated both in the nucleus and cytoplasm (Fig. 1B). Thus, ZNF217 was widely distributed in the ovaries, including follicles and corpus luteum.

### The expression of ovarian *ZNF217* was increased in OHSS rats while ovarian *TSP-1* decreased

**Figure 2:**
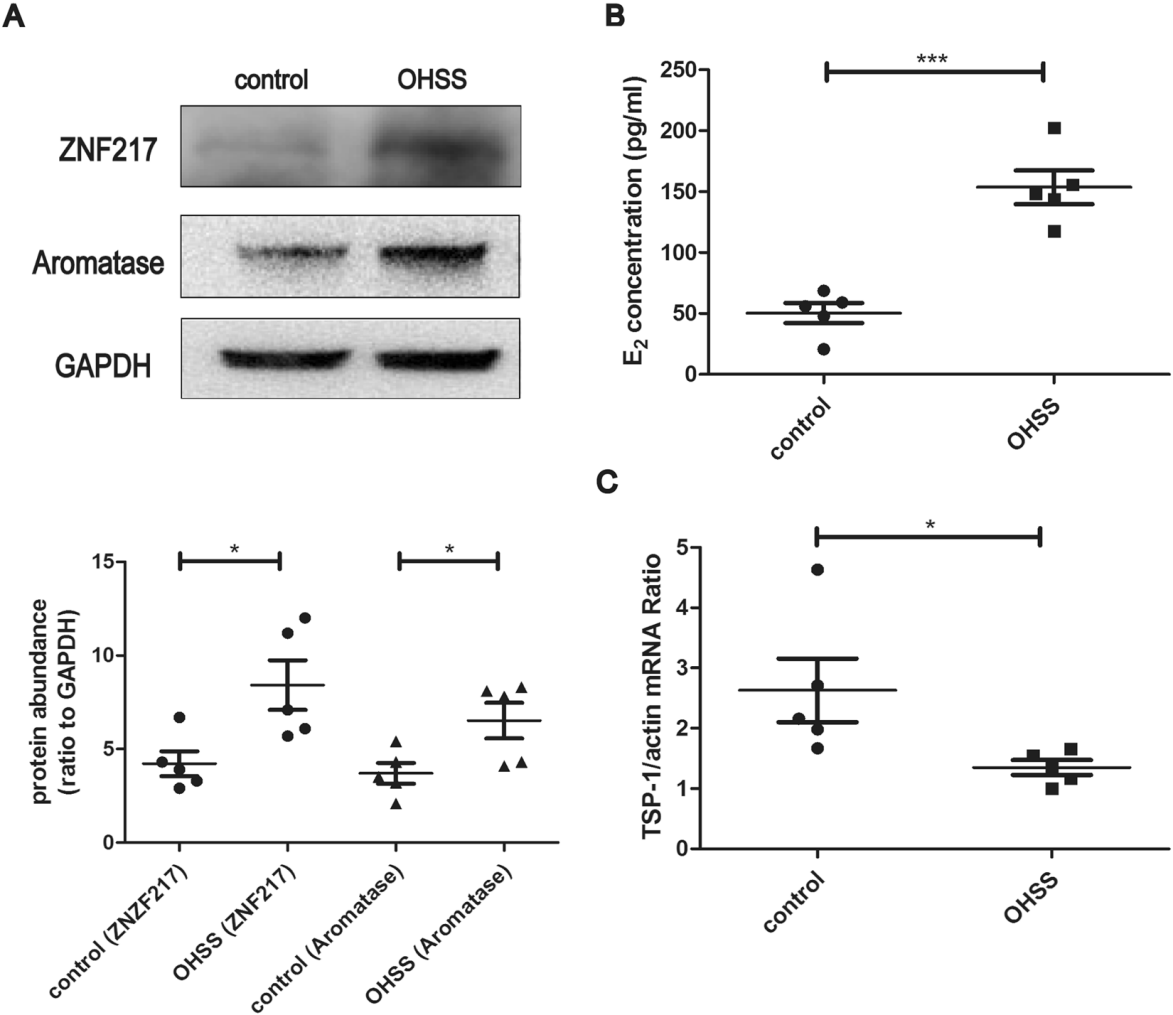
The expression of ovarian ZNF217 and ovarian TSP-1 in OHSS and control rats. (**A**) Western blot analysis of ovarian ZNF217 and aromatase both in control and OHSS rats (n = 5). Immunoblot signals were quantified by densitometry, and normalized with GAPDH. (**B**) Serum E_2_ concentration of control and OHSS rats before hCG administration (n = 5). (**C**) The expression of ovarian *TSP-1* mRNA in control and OHSS rats (n = 5). *P < 0.05, **P < 0.01. Data were expressed as mean ± SD. Western blots are the representative images.

The high ovarian size, ovarian weight, abdominal vascular permeability and ovarian VEGF of OHSS rats demonstrated that our OHSS model was successful (Supplemental Fig. 1). The expression of ovarian *ZNF217* of OHSS group was significantly higher than control group (p = 0.03) (Fig. 2A), being consistent with ovarian aromatase (key enzyme of E_2_ synthesis) (p = 0.04) (Fig. 2A) and serum E_2_ concentration (Fig. 2B). Moreover, ovarian *TSP-1* mRNA considerably decreased in the OHSS rats (Fig. 2C), which was contrary to the expression of *ZNF217*.

### Patients at high risk of OHSS showed an increased expression of *ZNF217* in the granulosa cells with a decreased TSP-1 in the follicular fluid

**Figure 3:**
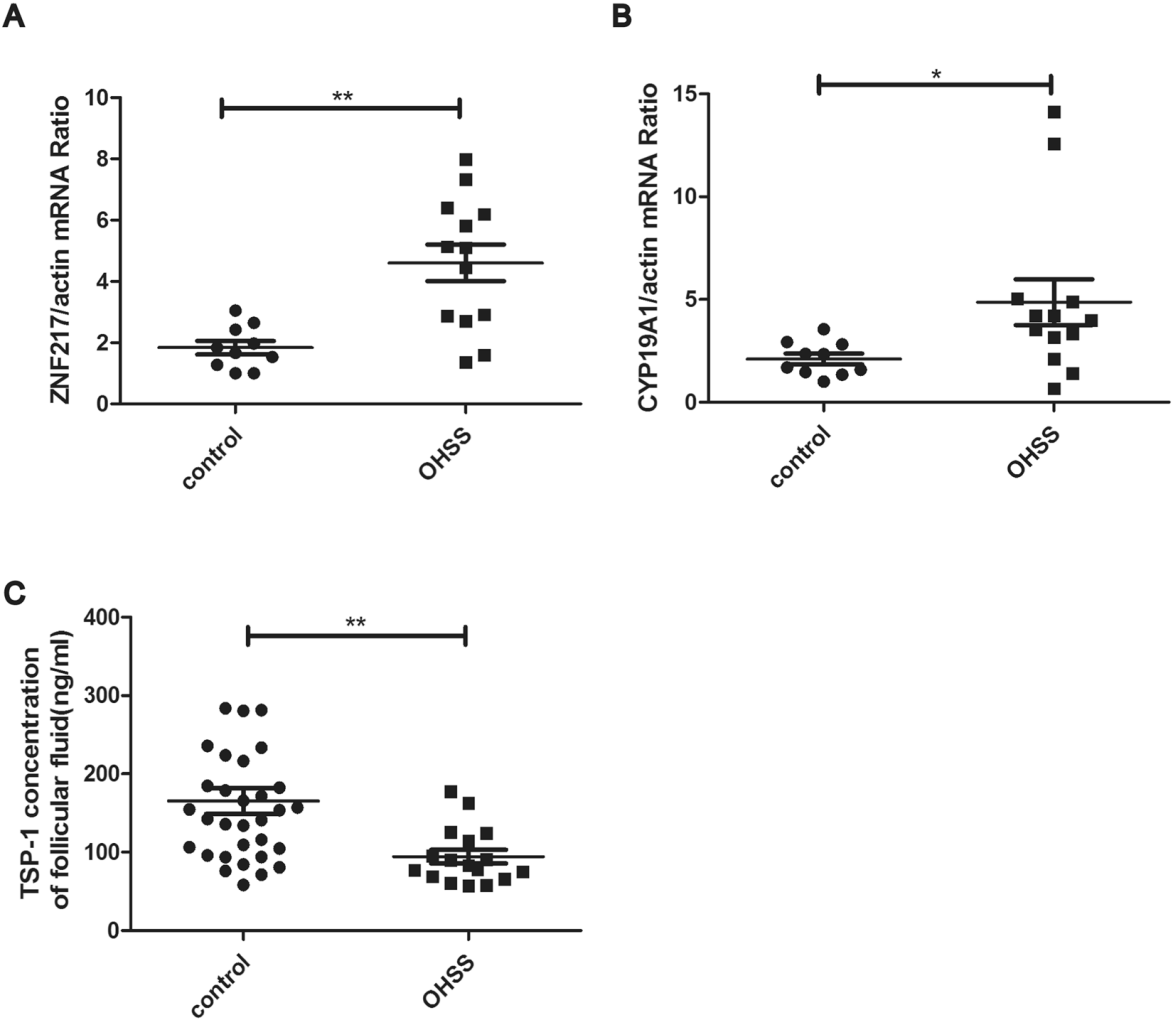
The expression of *ZNF217* and *TSP-1* of OHSS and control patients. (**A**) The expression of *ZNF217* mRNA in the ganulosa cells of high risk OHSS and control patients (control n = 10, OHSS n = 13) (t = −1.963, p = 0.064). (**B**) The expression of *CYP19A1* mRNA in the ganulosa cells of high risk OHSS and control patients (control n = 10, OHSS n = 13). (**C**) The TSP-1 concentration of the follicular fluid of the high risk OHSS and control patients (control n = 32, OHSS n = 17) (t = 3.796, p < 0.001). *P < 0.05, **P < 0.01.

Human granulosa cells and follicular fluid of patients at high risk of OHSS displayed the same trend as OHSS rat models. Both *ZNF217* mRNA (control n = 10, OHSS n = 13) (t = −4.172, p = 0.001) and *CYP19A1* mRNA (control n = 10, OHSS n = 13) (t = −2.259, p = 0.043) was highly expressed in the granulosa cells of high risk OHSS patients (Fig. 3A,B). Furthermore, the TSP-1 concentration of follicular fluid was significantly decreased in OHSS group compared with control group (control n = 32, OHSS n = 17) (t = 3.796, p < 0.001) (Fig. 3C). The clinic information of patients has been presented in Supplemental Table.

### ZNF217 positively regulated E_2_ synthesis through CREB1 and aromatase in KGN cells

**Figure 4:**
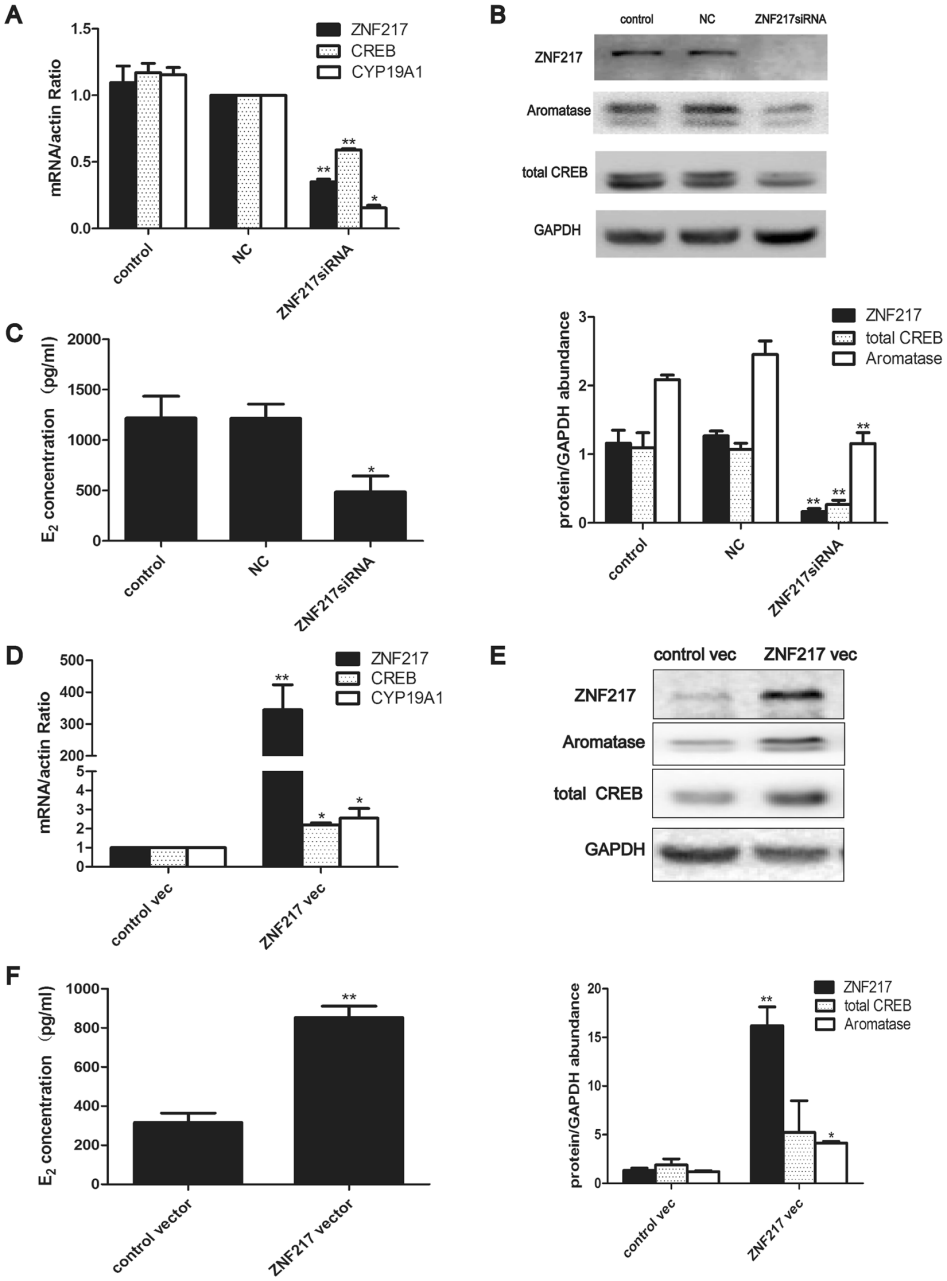
ZNF217 positively regulated E_2_ synthesis through CREB1 and aromatase in KGN cells. (**A**) The expression of *ZNF217, CREB1* and *CYP19A1* mRNA after *ZNF217* knock-down (n = 3). (**B**) Western blot analysis of ZNF217, total CREB and Aromatase after *ZNF217* knock-down (n = 3). Immunoblot signals were quantified by densitometry, and normalized with GAPDH. *ZNF217* siRNA was transfected into KGN cells for 48 h before the expression of downstream factors were detected. (**C**) E_2_ concentration in culture medium of KGN cells after *ZNF217* reduction (n = 3). KGN cells were pretreated with testosterone (10^−7^ mol/l) and E_2_ concentration in the culture medium was detected 3 h later. (**D**) The expression of *ZNF217*, *CREB1* and *CYP19A1* mRNA after *ZNF217* over-expression (n = 3). (**E**) Western blot analysis of ZNF217, total CREB and Aromatase after *ZNF217* over-expression (n = 3). Immunoblot signals were quantified by densitometry, and normalized with GAPDH. (**F**) E_2_ concentration in the culture medium of KGN cells after *ZNF217* over-expression (n = 3). *P < 0.05, **P < 0.01. Data were expressed as mean ± SD. Western blots are the representative images.

Both OHSS rat models and clinic human samples showed that ZNF217 was increased during OHSS onset, being consistent with high serum E_2_ concentration. Moreover, ZNF217 expressed in the granulosa cells of ovary which were the main source of E_2_ synthesis^20^. Thus, we supposed that ZNF217 might participate in E_2_ synthesis. KGN, a human granulosa cell line, has the expression of *ZNF217* and takes part in E_2_ synthesis^21^. Thus, KGN cells were chosen as the *in vitro* cell line to investigate the relationship between ZNF217 and E_2_ synthesis. *ZNF217* mRNA was reduced to less than 30 % compared with control group after specific siRNA treatment and the expression of CYP19A1 also significantly decreased both in mRNA and protein levels (mRNA p = 0.016, protein p = 0.007) (Fig. 4A,B). Moreover, decreased expression of total cAMP response element binding protein 1 (CREB1) was also observed after the reduction of *ZNF217* (mRNA p = 0.002, protein p = 0.001) (Fig. 4A,B). Thus, ZNF217 was involved in the regulation of CREB1, the upstream regulator of CYP19A1. The concentration of E_2_ in culture medium of KGN cells was also significantly reduced (p = 0.026) (Fig. 4C) after *ZNF217* knock-down. Therefore, ZNF217 was involved in E_2_ synthesis through positively regulating CREB1 and thereby CYP19A1. *ZNF217* vector transfection induced the over-expression of ZNF217 both in mRNA and protein level (Fig. 4D,E). Aromatase and total CREB1 also increased in line with the increase of ZNF217 both in mRNA and protein levels (aromatase p = 0.013, CREB1 p = 0.033) (Fig. 4D,E). Finally, the increased E_2_ concentration in the culture medium after *ZNF217* over-expression verified the positive regulation of ZNF217 to E_2_ synthesis in the granulosa cells (p = 0.001) (Fig. 4F).

### ZNF217 inhibited TSP-1 in KGN cells and then TSP-1 increased VP through CD36 and CD47 in human umbilical vein endothelial cells (HUVECs) and human aorta endothelial cells (HAECs)

**Figure 5:**
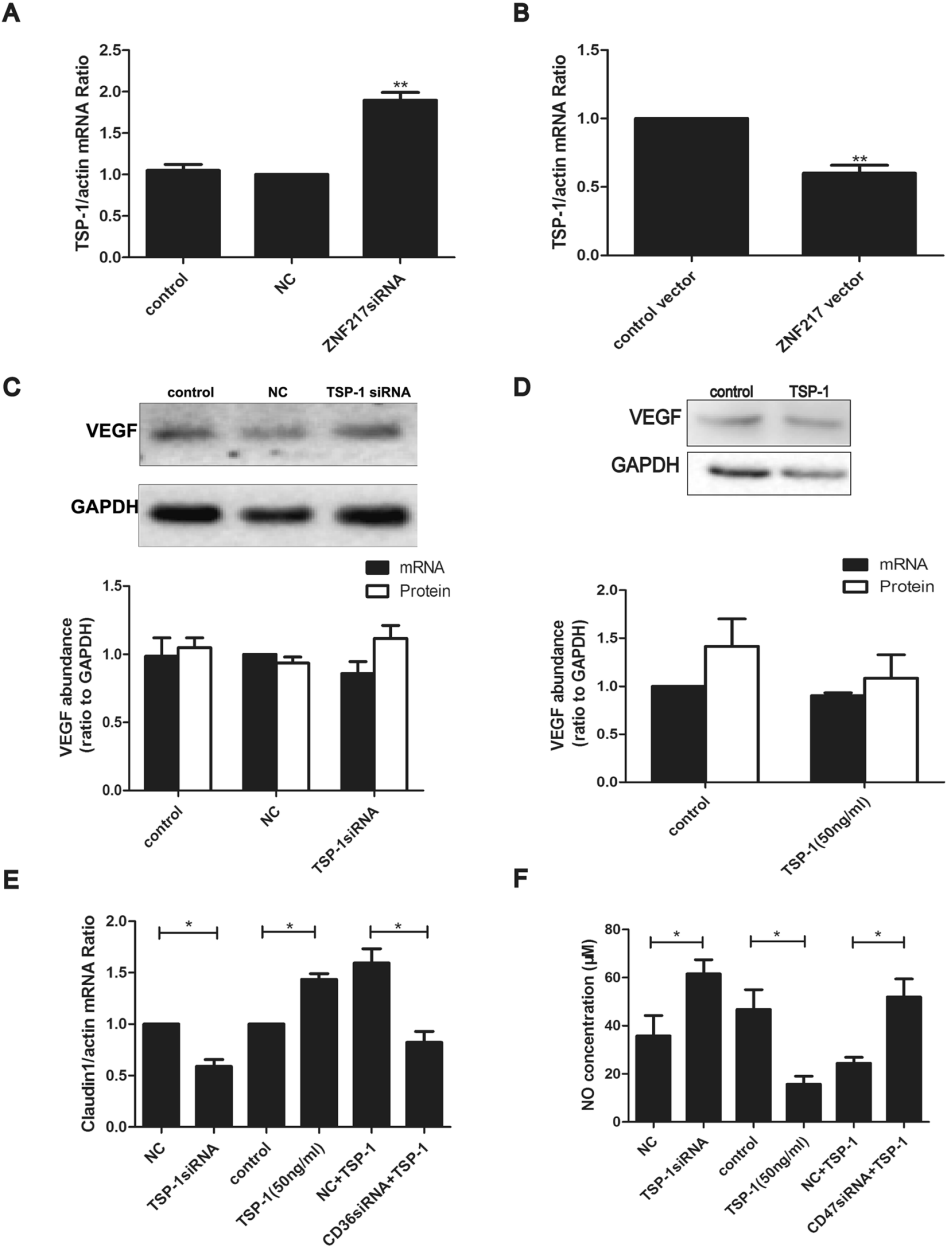
ZNF217 inhibited TSP-1 in KGN cells and then increased VP in HUVECs. (**A**) The expression of *TSP-1* mRNA after *ZNF217* knock-down in KGN cells. (**B**) The expression of *TSP-1* mRNA after *ZNF217* over-expression in KGN cells. (**C**) The expression of *VEGF* mRNA and western blot analysis of VEGF after *TSP-1* reduction in HUVECs. (**D**) The expression of *VEGF* mRNA and western blot analysis of VEGF after TSP-1 protein (50 ng/ml) treatment for 24 h in HUVECs. Immunoblot signals were quantified by densitometry, and normalized with GAPDH. E, The expression of *claudin1* mRNA level after *TSP-1* reduction, TSP-1 (50 ng/ml) treatment for 24 h and *CD36* knock-down in HUVECs. After *CD36* siRNA treatment for 48 h, HUVECs were treated with TSP-1 protein (50 ng/ml) for another 24 h. F, NO concentration of cell lysis of HUVECs after *TSP-1* knock-down, TSP-1 protein treatment for 24 h and *CD47* reduction. *P < 0.05, **P < 0.01. n = 3 separate experiments. Data were expressed as mean ± SD. Western blots are the representative images.

Significantly reduced ovarian TSP-1 was detected in both patients at high risk of OHSS and OHSS rat models, which displayed an opposite direction to ZNF217. Thus, TSP-1 may be negatively regulated by ZNF217 and play a role in OHSS onset. In KGN cells, *TSP-1* mRNA significantly increased after *ZNF217* reduction (p = 0.002) (Fig. 5A) while it was reduced after the over-expression of *ZNF217* (p = 0.002) (Fig. 5B). Therefore, ZNF217 negatively regulated *TSP-1* mRNA in granulosa cells. TSP-1 is a well-known suppressor of VEGF pathway and NO synthesis, implying its function in VP regulation. Both HUVECs and HAECs are endothelial cells which are involved in the regulation of VP. And TSP-1 of the follicular fluid could affect the biological behavior of the ovarian endothelial cells. Thus, HUVECs and HAECs were chosen to investigate the relationship between ZNF217, TSP-1 and VP. The expression of *VEGF* showed no significant change both after *TSP-1* reduction (Fig. 5C) and TSP-1 protein treatment (Fig. 5D) in HUVECs. Thus, TSP-1 didn’t directly regulate the expression of *VEGF* in HUVECs. However, *claudin1*, which is the downstream factor of VEGF pathway and regulates VP, significantly decreased after *TSP-1* reduction (p = 0.024) and increased after TSP-1 protein treatment (p = 0.017) (Fig. 5E). Moreover, the promotion function of TSP-1 to claudin1 was inhibited after *CD36* knock-down (p = 0.012) (Fig. 5E). It indicated the positive regulation of TSP-1 to claudin1 through CD36 in HUVECs. Meanwhile, NO, a well-known VP enhancer, significantly increased after *TSP-1* reduction (p = 0.027) and decreased after TSP-1 protein treatment (p = 0.025) (Fig. 5F). After *CD47* reduction, the inhibition of TSP-1 protein to NO was reversed (p = 0.013). Thus, TSP-1 inhibited NO synthesis after binding with CD47 in HUVECs.

There was no significant difference in NO concentration between the control group and the group treated with 50 ng/ml TSP-1 in HAECs though the decreasing tendency after the treatment consist with that in HUVECs (Supplemental Fig. 2B). Except the above result, other results of NO concentration and the mRNA expression of *claudin1* in HAECs, two makers of VP, were as the same as those in HUVECs (Supplemental Fig. 2A,B). However, TSP-1 inhibited the expression of *VEGF* in HAECs which was not observed in HUVECs (Supplemental Fig. 2C). We speculated that TSP-1 might affect VP via directly inhibiting the expression of *VEGF* and downstream NO and claudin1 in HAECs, while TSP-1 probably affected VP by altering the signaling pathway not the expression of VEGF in HUVECs. In a word, ZNF217 positively regulated VP through inhibiting TSP-1 and thereby inhibiting claudin1 as well as promoting NO synthesis.

## Discussion

*ZNF217* is a candidate oncogene which promoted the invasion and migration of breast and ovarian cancers^9, 22^. Recent studies indicated that *ZNF217* was a risk gene of PCOS which was vulnerable to OHSS onset^23^ and it enhanced the function of ERα in breast cancers^10^. Moreover, the increased ovarian *ZNF217* was detected in OHSS rats in our study. Based on previous results, we supposed that ZNF217 was closely related to OHSS. PMSG treatment promoted the development of follicles in rats so that most of follicles of control rats were antral follicles with follicullar antrum. In control groups, ZNF217 was mainly expressed in the granulosa cells of antral follicles, the main source of E_2_ synthesis^24^. Meanwhile, OHSS rats showed an increased serum E_2_ level, being consistent with ovarian ZNF217. Therefore, ZNF217 may be involved in the synthesis of E_2_ as well as the pathogenesis of OHSS.

In the ovary, local E_2_ acts in concert with the gonadotropins secreted from the anterior pituitary, especially follicle stimulating hormone (FSH), to provide for successful folliculogenesis. Aromatase, encoded by *CYP19A1*, is one of key enzymes of E_2_ synthesis and participates in the normal progress of the menstrual/estrous cycle^25^. The reduced E_2_ concentration and aromatase expression in KGN cells after *ZNF217* siRNA treatment indicated that ZNF217 positively regulated the synthesis of E_2_ through promoting aromatase. It is well accepted that FSH is the major inducer of aromatase activity in granulosa cells^26^. FSH stimulates the increase of cyclic adenosine monophosphate (cAMP) and the activation of cAMP-dependent protein kinase A (PKA)^27^. Then the activated PKA promotes the phosphorylation of cAMP-responsive element binding (CREB1) protein and triggers the binding of CREB1 to the promoter region of *CYP19A1* ^28^. Thus, the cAMP/PKA/CREB pathway is considered to be the primary signaling cascade through which the promoter of *CYP19A1* is regulated. Furthermore, CHIP-Seq in breast cancer cells indicated that *CREB1* was the target gene of ZNF217 and two binding sites in the promoter region of *CREB1* could be recognized by ZNF217^19^. Thus, we detected the relationship between ZNF217 and CREB1. CREB1 dramatically decreased after *ZNF217* knock-down in KGN cells. Over-expression of *ZNF217* verified the positive regulation of ZNF217 to E_2_ synthesis. Thus, ZNF217 promoted *CREB1* and then activated the synthesis of E_2_ through regulating aromatase. Apparently, ZNF217 was involved in the pathogenesis of OHSS.

TSP-1 is a potent VEGF pathway inhibitor while VEGF plays an indispensable role in OHSS onset. It is now apparent that the secreted protein TSP-1 inhibits NO production and NO signaling through endothelial cell nitric oxide synthase (e-NOS) after the activation of CD47. In addition, TSP-1 can be secreted by platelet and plays a mechanistic role in modulating thrombosis in the presence of von willebrand factor (VWF), which is also closely related to OHSS^29^. Thus, we assumed that TSP-1 played a role in OHSS onset. The significantly decreased TSP-1 both in the follicular fluid of patients at high risk of OHSS and the ovaries of OHSS rats certified the relationship between OHSS and TSP-1.

ZNF217 binds with ERα and enhanced the regulation of ERα to downstream factors while TSP-1 was negatively regulated by E_2_ in endometrial stromal cells. Moreover, OHSS patients showed an increased ZNF217 level, being contrary to TSP-1. Thus, ZNF217 may be involved in TSP-1 regulation. *TSP-1* mRNA significantly increased after *ZNF217* reduction and decreased after *ZNF217* over-expression in KGN cells. Therefore, TSP-1 was negatively regulated by ZNF217 in human granulosa cells. TSP-1 of granulosa cells could be secreted into follicular fluid and peripheral circulation to affect other cells of human tissues. VEGF pathway and NO can be regulated by TSP-1 and promote the increase of VP. Thus, TSP-1 exerts its functions about OHSS mainly in endothelial cells. Controversial results were identified about the regulation of TSP-1 to the expression of *VEGF* in HUVECs and HAECs. However, the results were consistent with previous reports which indicated that the effect of TSP-1 on VEGF differs in various kinds of cells. In the granulosa cells of mouse, TSP-1 has a direct inhibitory effect on VEGF by binding to the growth factor and internalizing it via low density lipoprotein receptor-related protein-1 (LRP-1)^30^. But in the choroidal endothelial cells of mouse, TSP-1 affected the expression of *VEGER* rather than VEGF directly^31^. Moreover, TSP-1 modulated VEGF signaling via CD36 and CD47 by affecting the phosphorylation of VEGFR2 in endothelial cells^16, 32^. Therefore, we detected the downstream factors of VEGF which were related to the regulation of VP in HUVECs and HAECs. Claudin1 is a tight junction protein of endothelial cells and it enhances cell junction and regulates VP. In our study, TSP-1 positively regulated claudin1 after combination with CD36. Moreover, TSP-1 also negatively regulated NO production, which was consistent with previous view that TSP-1 inhibited NO signaling after binding with CD47. Thus, ZNF217 increased VP through inhibiting TSP-1 and thereby promoting NO as well as inhibiting claudin1. In conclusion, the high-expression of ZNF217 in the granulosa cells induced a decreased TSP-1 in the follicular fluid. Then the decreased TSP-1 promoted the VP of ovarian endothelial cells and resulted in ovarian edema as well as ovarian enlargement, contributing to the pathogenesis of OHSS.

**Figure 6:**
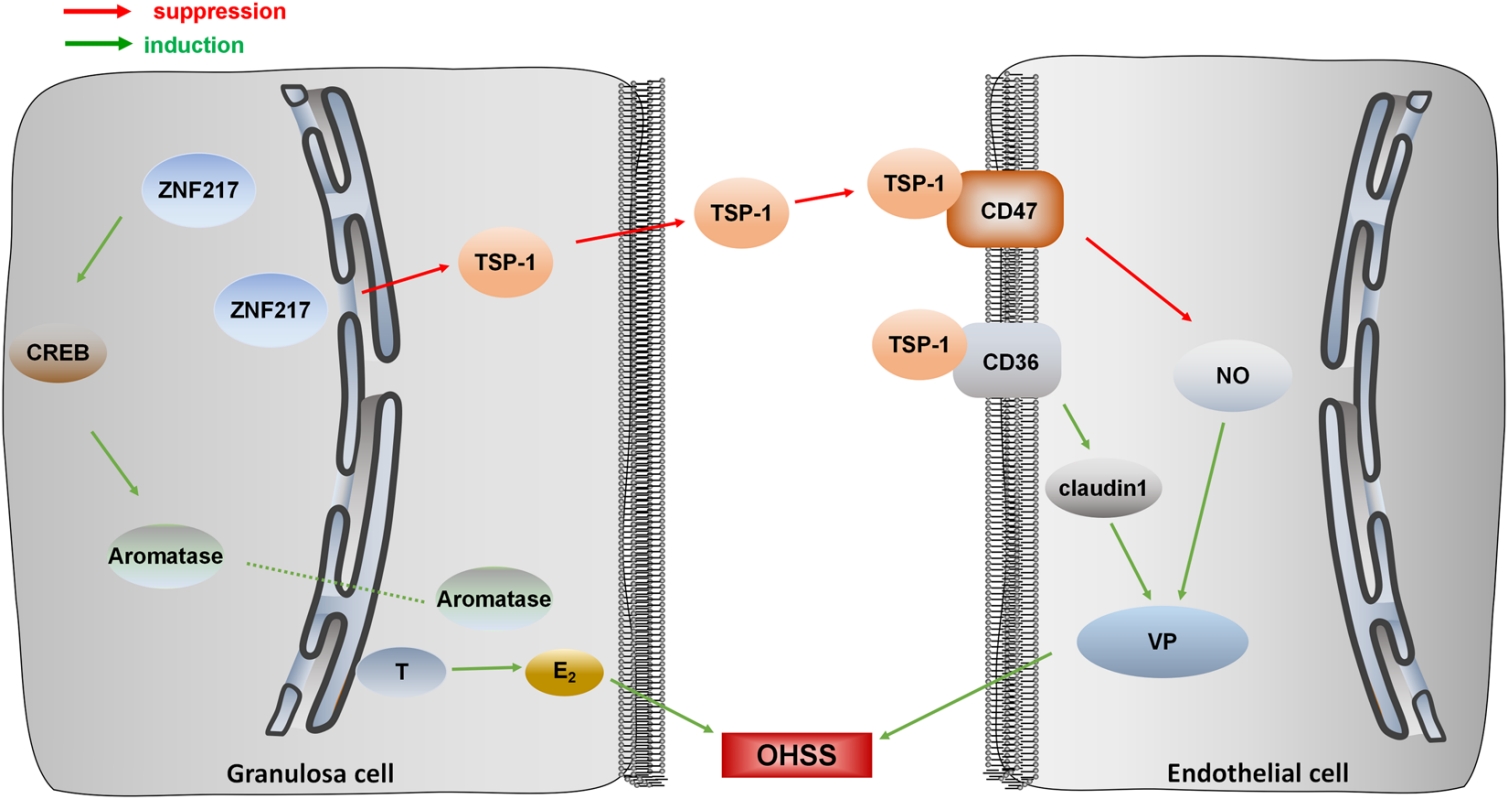
The proposed working model illustrates the mechanism underlying the promotion of ZNF217 to OHSS onset. High expression of *ZNF217* in human granulosa cells promoted the transcription of *CREB1* and thereby regulating aromatase. Then high E_2_ was secreted into serum and participated in the pathogenesis of OHSS. ZNF217 in granulosa cells also inhibited the expression of *TSP-1*, resulting in a low TSP-1 concentration in the follicular fluid. The decreased TSP-1 was secreted to follicular fluid and peripheral circulation to enhance NO synthesis and suppress claudin1, leading to a high VP in endothelial cells. Therefore, ZNF217 triggers OHSS onset by promoting E_2_ synthesis and inhibiting TSP-1. Green lines indicate promotion function while red lines suggest suppression.

Acoording to our study, high ZNF217 in human granulosa cells promoted CREB1 and aromatase, resulting in high E_2_ synthesis. Subsequently, E_2_ was secreted into serum and participated in the pathogenesis of OHSS. Meanwhile, ZNF217 of granulosa cells also inhibited the expression of TSP-1, resulting in a low TSP-1 concentration in follicular fluid. TSP-1 was secreted into the follicular fluid and peripheral circulation to exert its function. The decreased TSP-1 enhanced NO synthesis and NO signaling after binding with CD47^17^. Moreover, low level of TSP-1 also decreased its inhibition to VEGF pathway and suppressed claudin1 through binding with CD36, leading to a high VP (Fig. 6).

In conclusion, we clarified that ZNF217 triggered OHSS onset through promoting E_2_ synthesis and inhibiting TSP-1. Moreover, the increased ZNF217 and decreased TSP-1 provided potential targets for the treatment of OHSS in the future.

## Material and Methods

### Animal models

Immature 22-day-old female Wistar rats were fed ad libitum with a 12-hour light and 12-hour dark schedule and the rats were divided into two groups. OHSS group: Rats were given 10 IU PMSG (PROSPEC, East Brunswick, USA) by subcutaneous injection for 4 consecutive days to stimulate follicles development and then 30 IU hCG on the 5^th^ day to induce ovulation^33^ (n = 5). Control group: 24-day-old rats were given 10 IU PMSG and 10 IU hCG 2 days later to mimic a routine ovarian stimulation protocol. All the rats were killed by decapitation after hCG administration for 48 h.

All rat experimentation was conducted in accord with accepted standards of human animal care, as outlined in the Ethical Guidelines and the studies were approved by the Institutional Review Board of Ren Ji Hospital, School of Medicine, Shanghai Jiao Tong University.

### Clinical sample collection

Patients with high level of E_2_ (serum E_2_ level on the day of hCG administration was more than 6000 pg/ml) or more than 25 dominant follicles were identified as patients at high risk of OHSS. Follicles whose diameter was larger than 1.4 cm on the ovum retrieval day during IVF cycles were identified as dominant follicles. Cases with low level of E_2_ (< 4000 pg/ml) were chosen as the control group. Follicular fluid and granulosa cells from patients on the retrieval day were collected. The granulosa cells were purified with Ficoll-Paque^TM^ PLUS (GE-HealthCare Bio-Science, Uppsala, Sweden) and the granulosa cells were relatively pure. The follicular fluid of dominant follicle was centrifuged at a speed of 12000 rpm/min for 5 min before detection.

All the procedures were reviewed and approved by the Institutional Review Board of Ren Ji Hospital, School of Medicine, Shanghai Jiao Tong University (approval number 2015102306). The methods were carried out in accordance with the relevant guidelines and the informed consent was obtained from all subjects.

### Immunohistochemistry

The ovaries of OHSS and control rats were fixed with 4 % paraformaldehyde and embedded in paraffin. Then 5 μm sections were prepared which followed by deparaffinization and rehydration through a graded ethanol series. The tissue sections were blocked using rabbit serum for 1 h at room temperature and incubated with anti-ZNF217 antibody (Santa Cruz Biotechnology, Santa Cruz, CA, USA) (1:100) overnight at 4 °C in a dark chamber. After being washed with PBS, the sections were incubated with secondary antibody (1:400) for 1 h at room temperature and then the color reaction was visualized by exposure to diaminobenzidine (DAB) for 2 min. To test the specificity of immunocytochemical staining, separate tissue sections were exposed to preimmune serum instead of the primary antibody (negative control).

### Western Blot

Thirty μg of protein were loaded onto 10 % SDS gel coupled with loading buffer. Then the protein was transferred to nitrocellulose (NC) membrane and the nonspecific binding sites were blocked using 5 % non-fat dry milk. Then the NC membrane was incubated with diluted anti-ZNF217 antibody (Santa Cruz Biotechnology, Santa Cruz, CA, USA) (1:200), anti-total CREB antibody (Santa Cruz Biotechnology, Santa Cruz, CA, USA) (1:200), anti-aromatase antibody (Abcam, Cambridge, UK) (1:1000), anti-VEGF antibody (Santa Cruz Biotechnology, Santa Cruz, CA, USA) (1:200) at 4 °C for overnight. After washing with TBST, membrane was incubated with diluted peroxidase-conjugated secondary antibodies for 1 h at room temperature. At last, the protein signals were detected using ECL western blotting substrate.

### RNA extraction and real-time PCR

Total RNA from cells or tissues was extracted using animal total RNA isolation kit (FOREGENE, Chengdu, China) and then reversely transcripted into cDNA (TAKARA, Dalian, China).The expression of target genes were detected using real-time polymerase chain reaction (RT-PCR) and then we analyzed the results by ΔΔCt method. The housekeeping genes were *β-ACTIN*.

### Cell culture

KGN cells, HUVECs and HAECs were maintained in DMEM/F-12 medium (Gibco, Grand Island, NY), containing 10 % fetal bovine serum (Gibco, Grand Island, NY) and 1 % PSN (Gibco, Grand Island, NY). Cells were passaged every 3 days and incubated at 37 °C in a humidified atmosphere with 5 % CO_2_. Cells were digested and counted at 80–100 % before being seeded on six-well plates. HUVECs and HAECs were treated with TSP-1 protein (R&D systems, MN, USA) after seeded on plates for 24 h.

### Small interfering (si) RNA knocking down

Cells (2 × 10^5^) were seeded on six-well plates for 24 h and then the mixture of siRNA (50 pmol) and RNAiMAX (Invitrogen, Carlsbad, CA, USA) (9 μl) in OPTI-MEDIUM (250 μl) was added into each well. Then cells were further incubated for 48 h before testosterone treatment or the efficiency of knocking down was detected. The specific sequences of targeting genes were as follows:

*ZNF217* siRNA, sense, 5′-CGAUCAACGAGGUCGUCCATT-3.

anti-sense, 5′-UGGACGACCUCGUUGAUCGTT-3.

*TSP-1* siRNA, sense, 5′-GCGUGUUUGACAUCUUUGATT-3.

anti-sense, 5′-UCAAAGAUGUCAAACACGCTT-3.

*CD47* siRNA, sense, 5′-GACUUCUACAGGGAUAUUAdTdT-3.

anti-sense, 5′-UAAUAUCCCUGUAGAAGUCdTdT-3.

*CD36* siRNA, sense, 5′-GAGGAACUAUAUUGUGCCUTT-3.

anti-sense, 5′-AGGCACAAUAUAGUUCCUCTT-3.

Scrambled siRNA (NC), sense, 5′-UUCUCCGAACGUGUCACGUTT-3.

anti-sense, 5′-ACGUGACACGUUCGGAGAATT-3.

### Transfection of vectors in KGN cells with electroporation

KGN cells (6 × 10^6^) were mixed with 10 μg ZNF217 vector or p-enter vector (negative control) in OPTI-MEM and then the mixture was added into 2 mm gap cuvettes. Cells were electroporated at 170 V for 5 ms using a NEPA21 electroporator (Nepa Gene). After dilution with DMEM/F-12 containing 10 % fetal bovine serum, the cells were transferred onto three wells of six-well cell plate and incubated for 72 h before further treatment.

### E_2_ concentration measurement

KGN cells (2 × 10^5^) were seeded onto six-well plates. After siRNA treatment for 48 h or vector treatment for 72 h, we changed fresh culture medium containing certified charcoal stripped fetal bovine serum (Gibco, Grand Island, NY). Then testosterone (T) (10^−7^ mol/l) was added into cells and the culture medium was collected 3 h later. The culture medium was centrifuged at a speed of 12000 rpm/min for 5 min before the concentration of E_2_ was detected using Roche electrical chemiluminescence immunoassay after 40 times dilution.

The E_2_ concentration of rat serum before hCG administration was detected using Estradiol ELISA Kit (Cayman Chemical, Ann Arbor, MI).

### TSP-1 ELISA

The concentration of TSP-1 in human follicular fluid was detected using HUMAN TSP-1 ELISA KIT (R&D systems, MN, USA) without dilution. All the procedure was carried out according to standard protocol.

### NO concentration measurement

HUVECs and HAECs were lysed using cell and tissue lysis buffer for NO assay (Beyotime, Jiangsu, China) and then the NO concentration of lysate was detected using Griess reagent Kit (Beyotime, Jiangsu, China).

### Statistical analysis

Each experiment was performed for a minimum of three times. Results were displayed as the mean value ± standard deviation (SD). The differences between experimental and control groups were analyzed using one-way analysis of variance and unpaired student’s t-test of SPSS.

## Electronic supplementary material

Supplemental file
