# Supplementary material for: Zinc finger gene 217 (ZNF217) Promoted Ovarian Hyperstimulation Syndrome (OHSS) through Regulating E2 Synthesis and Inhibiting Thrombospondin-1 (TSP-1)
Source: Sci Rep. 2017 Jun 12;7:3245. doi: 10.1038/s41598-017-03555-6 (PMC5468349; doi:10.1038/s41598-017-03555-6)
Supplement: Supplementary file 1 — Supplemental file [file 41598_2017_3555_MOESM1_ESM.pdf]

Full title: Zinc finger gene 217 (ZNF217) Promoted Ovarian Hyperstimulation Syndrome (OHSS) through Regulating E_2 Synthesis and Inhibiting Thrombospondin-1 (TSP-1)

Authors: Junyu Zhai[1,2], Jiansheng Liu[1,2], Xiaoyue Cheng[1,2], Shang Li [1,2], Yan Hong[1,2], Kang Sun[1,2], Zi-Jiang Chen[1,2,3], Yanzhi Du[1,2]* , Weiping Li[1,2]*

Supplemental Table The clinic statistics of patients involved in the present study

	OHSS	control
age	29.79 ± 2.84	29 ± 2.85
FSH (IU/L)	6.85 ± 1.14	7.81 ± 1.89
LH (IU/L)	5.33 ± 1.55	5.19 ± 1.2
E2 (pg/ml)	43.99 ± 16	36.95 ± 14.27
T (nmol/l)	0.99 ± 0.49	0.94 ± 0.31
hCG (IU) **	4105 ± 1688	6062 ± 1692
E2 of hCG day (pg/ml) ***	7472 ± 1716	2002 ± 768
Follicle number ***	21 ± 8.84	9.62 ± 4.11

Supplemental Figure 1

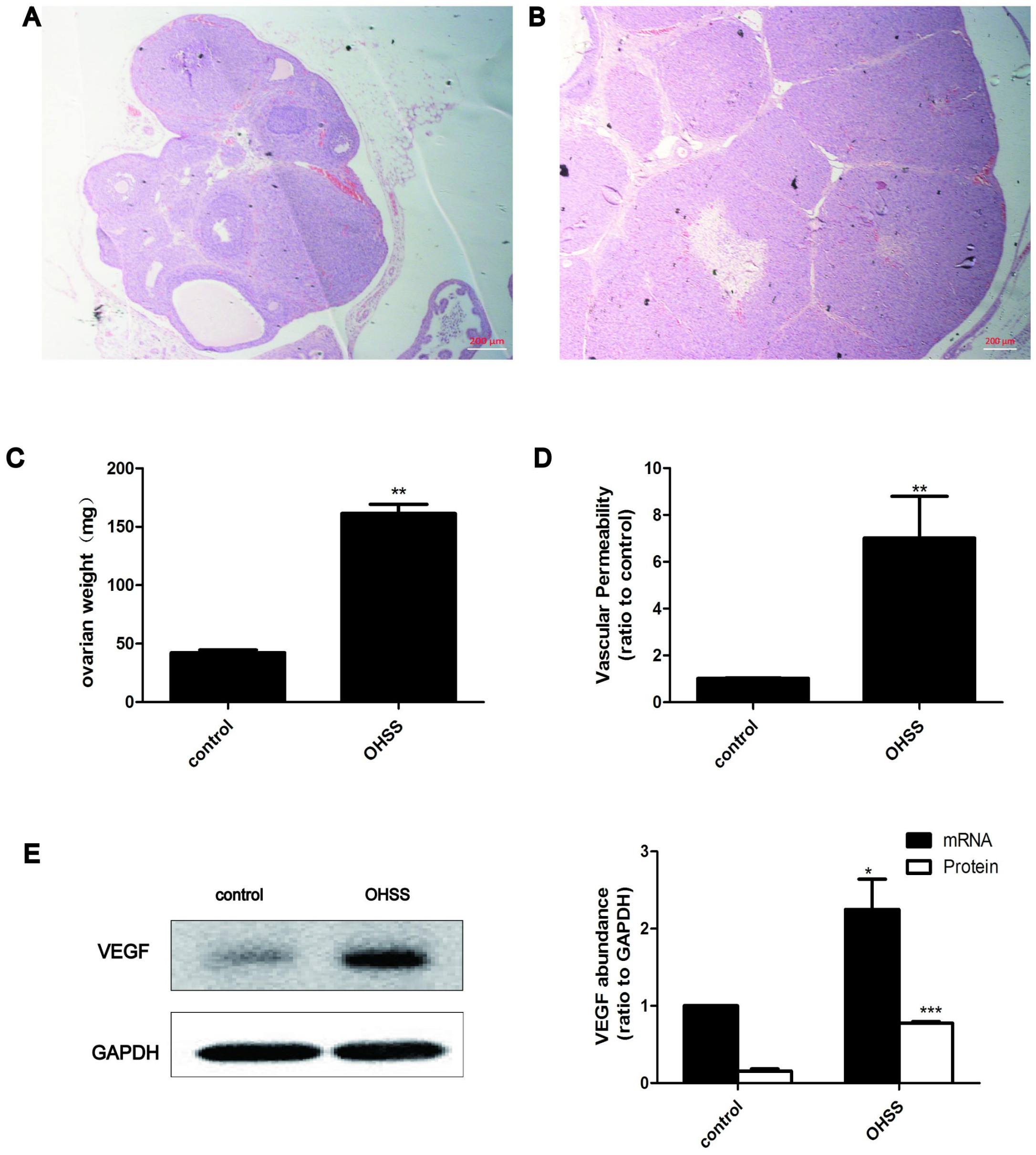

Supplemental Fig. 1. OHSS rat models were successful.

A, B, hematoxylin-eosin staining (HE) staining of control and OHSS rats ovaries. C, Ovarian weight of control and OHSS rats. D, Abdominal vascular permeability of control and OHSS rats. The OD value was used to represent the VP of rats and this figure showed the ratio of OHSS group to control group. E, The expression of ovarian *VEGF* mRNA and western blot analysis of VEGF in control and OHSS rats. Immunoblot signals were quantified by densitometry, and normalized with GAPDH. *P < 0.05, **P < 0.01, ***P=0.000, n=5 separate experiments. Western blots are the representative image.

Supplemental Figure 2

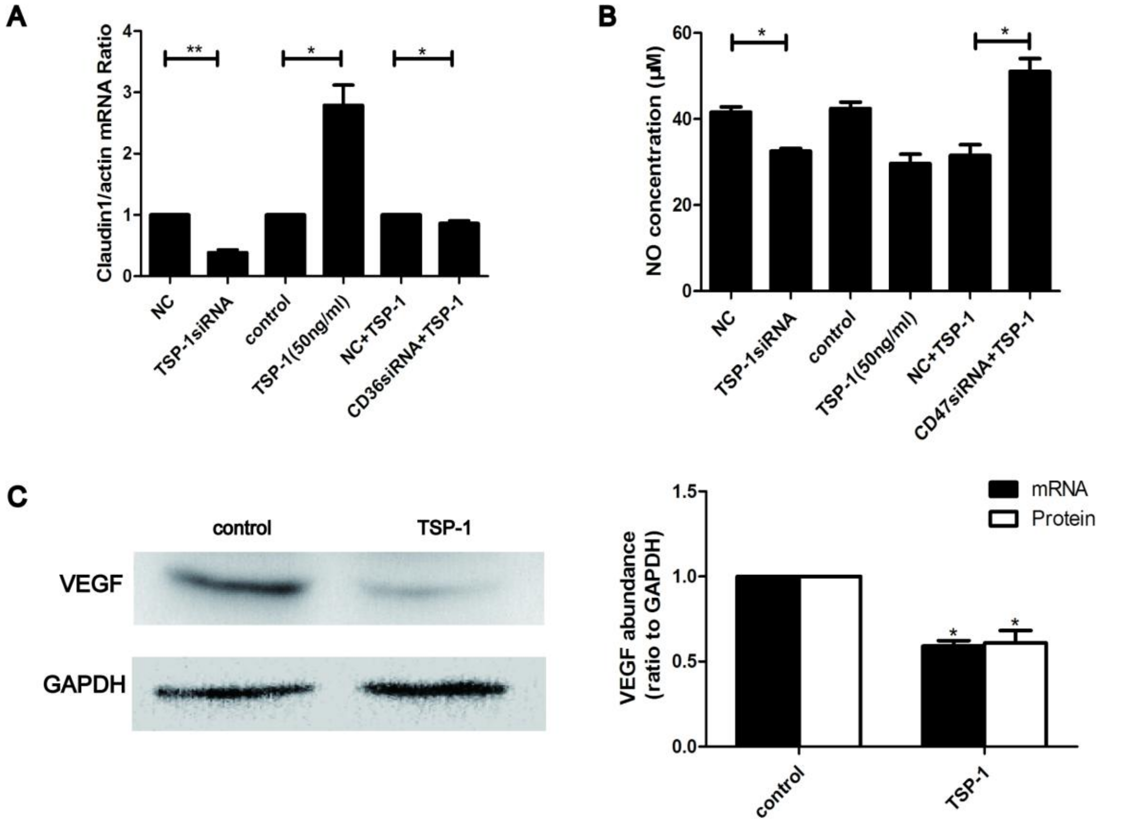

Supplemental Fig. 2. The regulation of TSP-1 to VP in HAECs.

A, The expression of *claudin1* mRNA level after *TSP-1* reduction, TSP-1 (50 ng/ml) treatment for 24 h and *CD36* knock-down in HAECs. After *CD36* siRNA treatment for 48 h, HAECs were treated with TSP-1 protein (50 ng/ml) for another 24 h. B, NO concentration of cell lysis of HAECs after *TSP-1* knock-down, TSP-1 protein treatment for 24 h and CD47 reduction. After *CD47* siRNA treatment for 48 h, HAECs were treated with TSP-1 protein (50 ng/ml) for another 24 h. C, The expression of *VEGF* mRNA and western blot analysis of VEGF after TSP-1 protein (50 ng/ml) treatment for 24 h in HAECs. Immunoblot signals were quantified by densitometry, and normalized with GAPDH. $*P < 0.05$, $**P < 0.01$. n=3 separate experiments. Data were expressed as mean ± SD. Western blots are the representative images.
